# Supplementary material for: Exploring ethical monitoring of physical activity behaviors among adults: a Smart Platform study operationalizing digital citizen science
Source: PeerJ. 2025 Aug 18;13:e19793. doi: 10.7717/peerj.19793 (PMC12369631; doi:10.7717/peerj.19793)
Supplement: Supplemental Information 1 [file peerj-13-19793-s001.docx]

Smart Adult Physical Activity Questionnaire

# Demographics

1. **What is your gender?**
   - (ID 1) Male
   - (ID 2) Female
2. **How old are you ………………**
3. **What is the highest level of education that you have attained?**
   - (ID1) Elementary school (some or completed)
   - (ID 2) Some secondary/high school
   - (ID 3) Completed secondary/high school
   - (ID 4) Some post-secondary (university or college)
   - (ID 5) Received university of college degree/diploma
4. **What best describes your employment status? Please check all that apply.**
   - (ID 1) Employed full-time
   - (ID 2) Employed full-time (self-employed)
   - (ID 3) Employed part-time
   - (ID 4) Employed part-time (self-employed)
   - (ID 5) Unemployed
   - (ID 6) Receiving social assistance
   - (ID 7) Receiving disability or retirement pension
   - (ID 8) Student
   - (ID 9) Other (i.e. seasonal temporary)

# Health Motivation

1. **I undertake physical activity to improve my cardiovascular fitness.**
   - (ID 1) Strongly disagree
   - (ID 2) Disagree
   - (ID 3) Neutral
   - (ID 4) Agree
   - (ID 5) Strongly agree
   - (ID 6) Does not apply
2. **I undertake physical activity to maintain physical health.**
   - (ID 1) Strongly disagree
   - (ID 2) Disagree
   - (ID 3) Neutral
   - (ID 4) Agree
   - (ID 5) Strongly agree
   - (ID 6) Does not apply

# Recreational Motivation

1. **I undertake physical activity because it's fun.**
   - (ID 1) Strongly disagree
   - (ID 2) Disagree
   - (ID 3) Neutral
   - (ID 4) Agree
   - (ID 5) Strongly agree
   - (ID 6) Does not apply

# Environmental Factors

1. **I have facilities/equipment to exercise in my home.**
   - (ID 1) Strongly disagree
   - (ID 2) Disagree
   - (ID 3) Neutral
   - (ID 4) Agree
   - (ID 5) Strongly agree
   - (ID 6) Does not apply
2. **Please rate the following set of statements. I have space to exercise in my home.**
   - (ID 1) Strongly disagree
   - (ID 2) Disagree
   - (ID 3) Neutral
   - (ID 4) Agree
   - (ID 5) Strongly agree
   - (ID 6) Does not apply
