# Supplementary material for: Exploring ethical monitoring of physical activity behaviors among adults: a Smart Platform study operationalizing digital citizen science
Source: PeerJ. 2025 Aug 18;13:e19793. doi: 10.7717/peerj.19793 (PMC12369631; doi:10.7717/peerj.19793)
Supplement: Supplemental Information 2 — Each coefficient represents the estimated association between endorsing a given physical activity (PA) motivation (i.e., “agree” vs. “disagree,” the latter serving as the reference category) and the corresponding measure of PA. Results are presented for two models—Model 1 (retrospective PA) and Model 2 (prospective PA)—across the overall sample as well as stratified by gender (male and female). Numbers in parentheses indicate the 95% confidence intervals for each estimate, and significance levels are denoted as follows: ∗p < .05, ∗∗p < .01, and ∗∗∗p < .001. The “Ref” entries mark the baseline (disagree) category for each independent variable, and the Constant represents the model intercept. [file peerj-13-19793-s002.docx]

**Appendix 1:** Sensitivity analyses showing associations between retrospective and prospective measured PA and social, and health motivations of PA in the overall and gender sample.

|  | **Overall** | | **Male** | | **Female** | |
| --- | --- | --- | --- | --- | --- | --- |
|  | **Model 1: retrospective PA** | **Model 2: prospective PA** | **Model 1: retrospective PA** | **Model 2: prospective PA** | **Model 1: retrospective PA** | **Model 2: prospective PA** |
| **Independent Variables** |  |  |  |  |  |  |
| Undertake PA because it’s fun – disagree | **Ref** | **Ref** | **Ref** | **Ref** | **Ref** | **Ref** |
| Undertake PA because it’s fun – agree | 0.573*** (0.216, 0.930) | 1.563*** (0.745, 2.381) | 0.841** (0.107, 1.575) | 0.442 (-1.246, 2.129) | 0.602*** (0.177, 1.028) | 1.960*** (0.964, 2.957) |
| Has facilities/equipment to exercise at home – disagree | **Ref** | **Ref** | **Ref** | **Ref** | **Ref** | **Ref** |
| Has facilities/equipment to exercise at home – agree | 0.120 (-0.272, 0.512) | 0.432 (-0.467, 1.331) | 0.047 (-0.664, 0.758) | 1.203 (-0.432, 2.837) | 0.232 (-0.249, 0.713) | 0.214 (-0.911, 1.340) |
| To improve cardiovascular fitness – disagree | **Ref** | **Ref** | **Ref** | **Ref** | **Ref** | **Ref** |
| To improve cardiovascular fitness – agree | 0.045 (-0.522, 0.612) | -0.233 (-1.533, 1.068) | 0.531 (-0.389, 1.451) | -0.372 (-2.486, 1.742) | -0.338 (-1.090, 0.414) | -0.208 (-1.967, 1.551) |
| Space to exercise at home – disagree | **Ref** | **Ref** | **Ref** | **Ref** | **Ref** | **Ref** |
| Space to exercise at home – agree | 0.287 (-0.121, 0.695) | -0.119 (-1.054, 0.815) | 0.240 (-0.522, 1.001) | -0.455 (-2.206, 1.295) | 0.191 (-0.309, 0.690) | -0.153 (-1.322, 1.017) |
| Maintain physical health – disagree | **Ref** | **Ref** | **Ref** | **Ref** | **Ref** | **Ref** |
| Maintain physical health – agree | 0.588 (-0.206, 1.381) | 1.345 (-0.473, 3.163) | 1.252* (-0.138, 2.643) | 1.622 (-1.573, 4.818) | 0.299 (-0.690, 1.288) | 1.016 (-1.298, 3.330) |
| Constant | 4.190*** (3.310, 5.070) | 1.585 (-0.432, 3.602) | 3.356*** (1.783, 4.930) | 2.162 (-1.456, 5.780) | 4.657*** (3.574, 5.741) | 1.507 (-1.029, 4.042) |
| Observations^β^ | 126 | 126 | 43 | 43 | 83 | 83 |
