# Supplementary figures and images for: Exploring ethical monitoring of physical activity behaviors among adults: a Smart Platform study operationalizing digital citizen science
Source: PeerJ. 2025 Aug 18;13:e19793. doi: 10.7717/peerj.19793 (PMC12369631; doi:10.7717/peerj.19793)

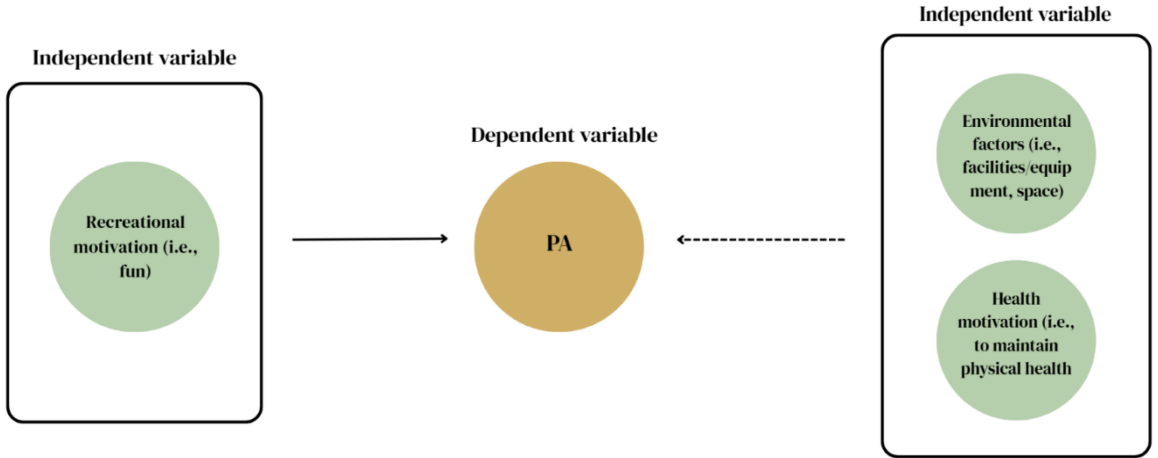

Supplement: Supplemental Information 3 — Solid line represents a significant association. Dotted line represents no significant association. [file peerj-13-19793-s003.pdf]
