# Supplementary material for: Exploring ethical monitoring of physical activity behaviors among adults: a Smart Platform study operationalizing digital citizen science
Source: PeerJ. 2025 Aug 18;13:e19793. doi: 10.7717/peerj.19793 (PMC12369631; doi:10.7717/peerj.19793)
Supplement: Supplemental Information 4 — Solid line represents a signficant association. Dotted line represents no significant association. [file peerj-13-19793-s004.pdf]

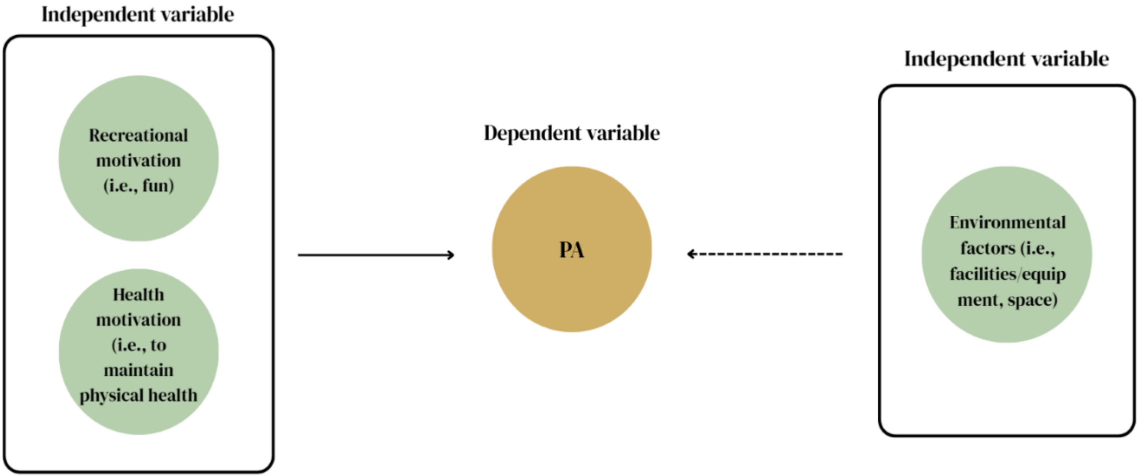
Independent variable
Recreational motivation (i.e., fun)
Health motivation (i.e., to maintain physical health
Dependent variable
PA
Independent variable
Environmental factors (i.e., facilities/equipment, space)
